# Cancer Research

**Published:** 1905-07-08

**Authors:** 


### CANCER RESEARCH.

At the annual meeting of the General Committee of the Imperial Cancer Research Fund, held at Marlborough House on Wednesday, under the presidency of his Royal Highness the Prince of Wales, reports were presented from the secretary, from the general superintendent, and from the honorary treasurer. The report of the superintendent, Dr. Bashford, was mainly a summary of two scientific reports just published by the authority of the Executive Committee, and dealing respectively with the statistical investigation of cancer and with the growth of cancer under natural and experimental conditions. With reference to the former subject, Dr. Bashford mentions that a first series of reports from India includes 146 cases in natives living on a vegetable diet, 137 in natives whose food was mainly flesh, and 222 in natives living on a mixed diet. He sketches the nature of the general statistical inquiries which have been undertaken, and points out the difficulties which stand in the way of obtaining sufficient and trustworthy data. Much additional evidence has accumulated in support of the view that the number of cases of cancer, wherever occurring, depends to an important extent upon the number of aged individuals coming under observation, both in different races of mankind and in animals. It is not yet possible to determine statistically whether cancer is really increasing as the increase in recorded cases would imply.

A very large number of inoculation experiments have been performed upon mice, and have been attended with a degree of success which only became apparent after the experiments had been systematically continued for almost two years. It has been proved that all the features of cancer can be reproduced experimentally, a result "the most important and far-reaching of those hitherto obtained." The true nature of some features and their relations to one another have been revealed by experiment. Certain fundamental features have also been separated from others of subsidiary importance, and hitherto unsuspected properties have been brought to light.

We may summarise some of the more important of the results attained by saying that the disease in mice has been proved to be in all respects like cancer in the human subject, and that it is readily inoculable from mouse to mouse. The malignant new growth is an independent organism except in so far as it is dependent for its nutrition upon the mouse in which it is growing. It is, indeed, a sort of parasite, consisting of special cells, but the stroma in which these cells are supported, and the vascular system by which they are nourished, are supplied by the host. When a fragment of cancer is introduced into the body of a healthy mouse, any portions of stroma or of vascular tissue which may be included perish, while the essential and isolated cells remain and increase. A cancerous growth perishes, of course, with the death of the host, but may be preserved alive indefinitely by previous inoculations into other hosts, and thus seems to have no term of existence of its own. Under artificial propagation a single mouse tumour has produced an amount of tissue sufficient to yield a giant mouse as large as a St. Bernard dog, or several thousand adult mice. In the amount which suffices to kill the individual primarily affected only an infinitesimal fraction of the power of cell division residing in malignant tissues, as such, is manifested, and hence a single case gives an imperfect conception of the process concerned. Old age is most constantly associated with the origin of cancerous proliferation, but it has been possible to show that the enormous cell-division which endangers life is not dependent upon constitutional changes in the animal primarily attacked. Under artificial propagation it proceeds as well in young mice as in old ones. In experimental transmission the tissues of the new hosts do not acquire cancerous properties, and it has been definitely proved that no analogy exists between cancer and any known form of infective or contagious disease, so that cancer is not caused by the parasite of any such disease entering the body from without. Future work must be directed to ascertaining why cancer arises *de novo* in each individual attacked, why it is more intimately bound up with the later than with the earlier stages in the life of the individual, and what change or changes the cells of normal tissues undergo in acquiring cancerous properties. The work done has thrown fresh light on the well-known features of the disease, and points to the necessity of investigating the minute cell processes which are responsible for the ceaseless cell-division and growth of cancer when propagated artificially. The results already attained are such as to justify great hopefulness for the future.

The report of the honorary treasurer announces that the amount of the fund is now £75,000, or £25,000 less than it was desired to obtain, and he asks earnestly for fresh contributions.

